# Origins of Albino and Hooded Rats: Implications from Molecular Genetic Analysis across Modern Laboratory Rat Strains

**DOI:** 10.1371/journal.pone.0043059

**Published:** 2012-08-16

**Authors:** Takashi Kuramoto, Satoshi Nakanishi, Masako Ochiai, Hitoshi Nakagama, Birger Voigt, Tadao Serikawa

**Affiliations:** 1 Institute of Laboratory Animals, Graduate School of Medicine, Kyoto University, Sakyo-ku, Kyoto, Japan; 2 National Cancer Center Research Institute, Chuo-ku, Tokyo, Japan; Ecole Normale Supérieure de Lyon, France

## Abstract

Albino and hooded (or piebald) rats are one of the most frequently used laboratory animals for the past 150 years. Despite this fact, the origin of the albino mutation as well as the genetic basis of the hooded phenotype remained unclear. Recently, the albino mutation has been identified as the Arg299His missense mutation in the Tyrosinase gene and the hooded (*H*) locus has been mapped to the ∼460-kb region in which only the *Kit* gene exists. Here, we surveyed 172 laboratory rat strains for the albino mutation and the hooded (*h*) mutation that we identified by positional cloning approach to investigate possible genetic roots and relationships of albino and hooded rats. All of 117 existing laboratory albino rats shared the same albino missense mutation, indicating they had only one single ancestor. Genetic fine mapping followed by *de novo* sequencing of BAC inserts covering the *H* locus revealed that an endogenous retrovirus (ERV) element was inserted into the first intron of the *Kit* gene where the hooded allele maps. A solitary long terminal repeat (LTR) was found at the same position to the ERV insertion in another allele of the *H* locus, which causes the so called Irish (*h^i^*) phenotype. The ERV and the solitary LTR insertions were completely associated with the hooded and Irish coat patterns, respectively, across all colored rat strains examined. Interestingly, all 117 albino rat strains shared the ERV insertion without any exception, which strongly suggests that the albino mutation had originally occurred in hooded rats.

## Introduction

The rat (*Rattus norvegicus*) was the first mammalian species domesticated for scientific research with work dating back to before 1850. Since that time, the rat has a leading role in various research fields, such as physiology, pharmacology, neurosciences, genetics, and medical sciences. Among the rat strains available now, the albino and piebald (hooded) are most common, which is the same situation as in the earliest days when rats were firstly used for scientific research.

Albino rats have played a pioneering role in animal experimentation since its inception. Gregor Mendel reported his famous laws on ‘Mendelian inheritance’ in 1866. Hugo Crampe was the first scientist to confirm the validity of these laws in animals using some 15,000 white, grey, black, and piebald rats between 1877 and 1885 [1]. These albino rats were in fact the first animals to be domesticated for the purpose of scientific research.

The “hooded” phenotype is also one of the oldest mutations in the rat. Hooded rats have a pattern in which the entire ventral surface is white. Dorsally pigmentation is limited to the head and shoulders (the “hood”) and a mid-dorsal stripe extending back to the tip of the tail. In addition to this hooded (*h)*, other modifiers such as Irish and notch alleles are known in the so named *H* locus [2]. The Irish (*h^i^*) causes a white spot on the belly between and behind the front legs. Notch (*h^n^*) rats have a white body with pigmented fur on the sides of the head. The wild-type allele against the *h*, *h^i^*, or *h^n^* is called “self” (*H*). Self animals are pigmented all over.

Despite their undisputed importance to scientific progress, the origin of albino and hooded rats has never been clearly determined. The question remains unanswered, is there only one single spontaneous mutation that has been transferred and bred for more than 100 years into hundreds existing albino rat strains? Or, did several different mutations such as those in mice [3] contribute to the albino phenotype that is now seen in millions of laboratory rodents around the world?

Henry H Donaldson at the Wistar Institute in Philadelphia wrote in 1915: “The Norway rat, *Mus norvegicus* (now *Rattus norvegicus*), is the one mammal easily obtainable both wild and as a domesticated form. This latter is represented by either the albino or the pied rats so common in our laboratories. We do not know whether the common albino variety had a single or multiple origins, or whether the colonies found in Europe are directly related to those now existing here” [4].

A deeper look into the historic records on rodents and a detailed molecular genetic analysis of the genetic patterns of all available coat color variations in rats are the measures of choice for solving these questions. The molecular nature of the albino rat has been identified as Arg299His missense mutation in the rat tyrosinase (*Tyr*) gene [5]. The Arg299His mutation has already been described in humans affected by ocular cutaneous albinism 1A (OCA1A) without any tyrosinase activity and consequential complete lack of pigmentation [6]. The hooded (*H*) locus was mapped to the rat Chr 14 [7]. Recently, Torigoe et al. mapped the *H* locus to the ∼460-kb region in which only the *Kit* gene existed but did not found any mutations in the *Kit* coding region of the hooded LEA rats [8].

The aim of this study is to clarify and characterize the origin and nature of albino and hooded mutations using molecular genetic approaches and combine the findings with historical records. The albino related question was addressed by genotyping the *Tyr* gene mutation Arg299His for 172 laboratory strains from different regions of the world. In parallel, we confirmed the mapping of the hooded locus to the *Kit* gene on the rat Chr 14 by genetic studies and subsequently identified genetic variations in the *Kit* gene that are unique for hooded rats.

## Results

### Genotyping of the Albino Allele

Preliminary sequencing of several albino strains confirmed the Arg299His mutation. Subsequent screening across all 172 strains examined was performed by direct digestion of the PCR product with the specific *Sna*BI restriction endonuclease to genotype the Arg299His mutation in the rat *Tyr* gene. All 55 colored rat strains had the wild-type allele (299Arg) and all of the 117 albino rat strains had the missense mutation (299His) (**Table S1**).

### Fine Mapping of the Rat Hooded (H) Locus

**Figure 1:**
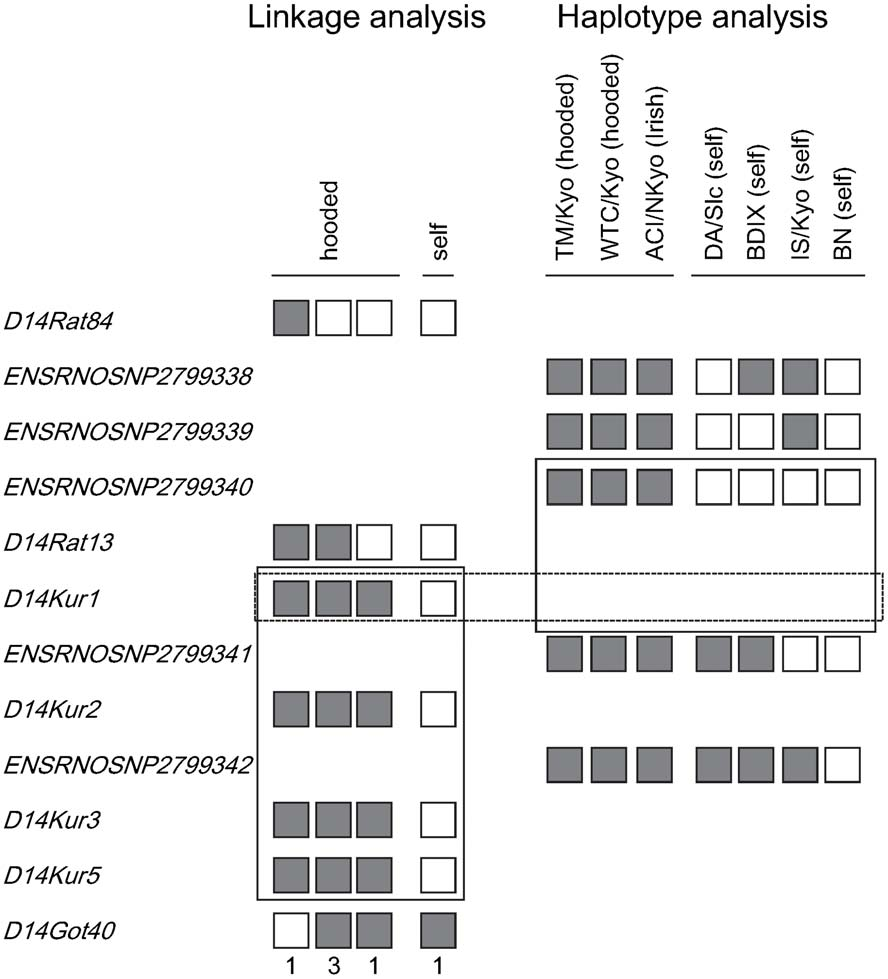
Linkage and haplotype mapping of the hooded (H) locus. Left: Haplotypes of backcross progeny carrying the recombinant chromosome between D14Rat84 and D14Got40. The grey boxes represent rats homozygous for genetic markers, while the white boxes represent rats heterozygous for genetic markers. The coat pattern phenotypes of the progeny and the number of the progeny for each haplotype are described above and below the haplotypes, respectively. The H locus was narrowed down from D14Rat13 to D14Got40 (boxed with a solid line). Right: SNP haplotypes around the H locus in representative inbred rat strains. Two hooded rat strains and one Irish rat strain shared the identical haplotype of the genomic region between ENSRNOSNP2799338 and ENSRNOSNP2799342. The genomic region that was shared among the four self strains was narrowed down from ENSRNOSNP2799339 to ENSRNOSNP2799341 (boxed with a solid line). The linkage and haplotype analyses demonstrated that the hooded locus was mapped to the region defined by D14Rat13 and ENSRNOSNP2799341 (boxed with a dashed line).

**Figure 2:**
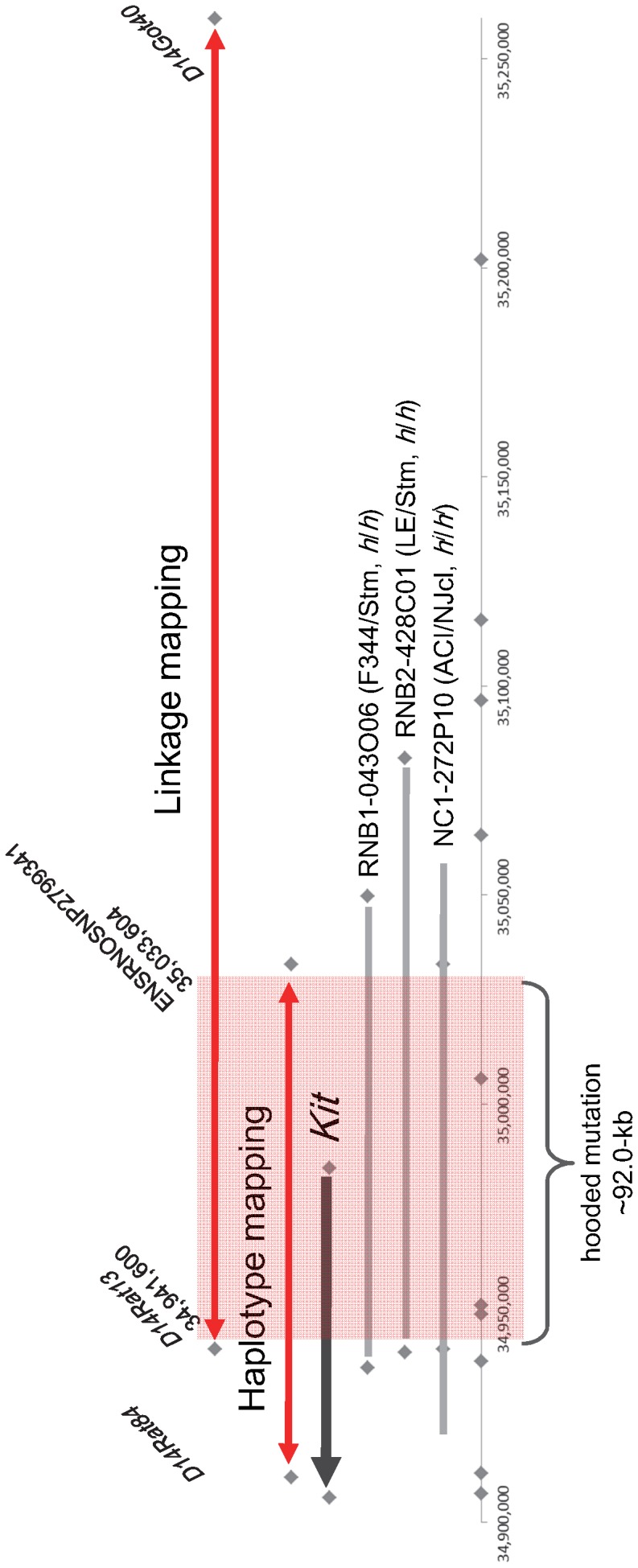
Physical position of the hooded mutation. The hooded mutation spanned to the 92 kb region defined by D14Rat13 and ENSRNOSNP2799341 (boxed in red). This region contains the upstream region and five exons of the rat Kit gene (black arrow). The hooded critical region was covered by BACs that contain F344/Stm, LE/Stm and ACI/NJcl genomic DNAs. Grey bars represent the regions covered by BACs. Grey squares represent positions of the SSLP or SNP makers examined.

Torigoe et al. mapped the *H* locus between *D14Rat84* and *D14Got40* [8]. Genotyping of the backcross progeny for both SSLP markers revealed six animals carrying the recombinant chromosome between *D14Rat84* and *D14Got40*. By using five SSLP markers, we narrowed the *H* locus down to between *D14Rat13* (position 34,941 kb) and *D14Got40* (position 35,259 kb). Haplotype analysis revealed that the *H* locus spanned from *ENSRNOSNP2799339* (position 34,910 kb) to *ENSRNOSNP2799341* (position 35,033 kb) (**Fig. 1**). These findings clearly indicated that the *H* locus spanned about 92 kb defined by *D14Rat13* and *ENSRNOSNP2799341* (**Fig. 2**). This hooded critical region contained the five exons (1 to 5) of the *Kit* gene and the 48.8 kb genomic region upstream of the *Kit* gene.

### Endogenous Retrovirus Sequences in the Rat Kit Gene

**Figure 3:**
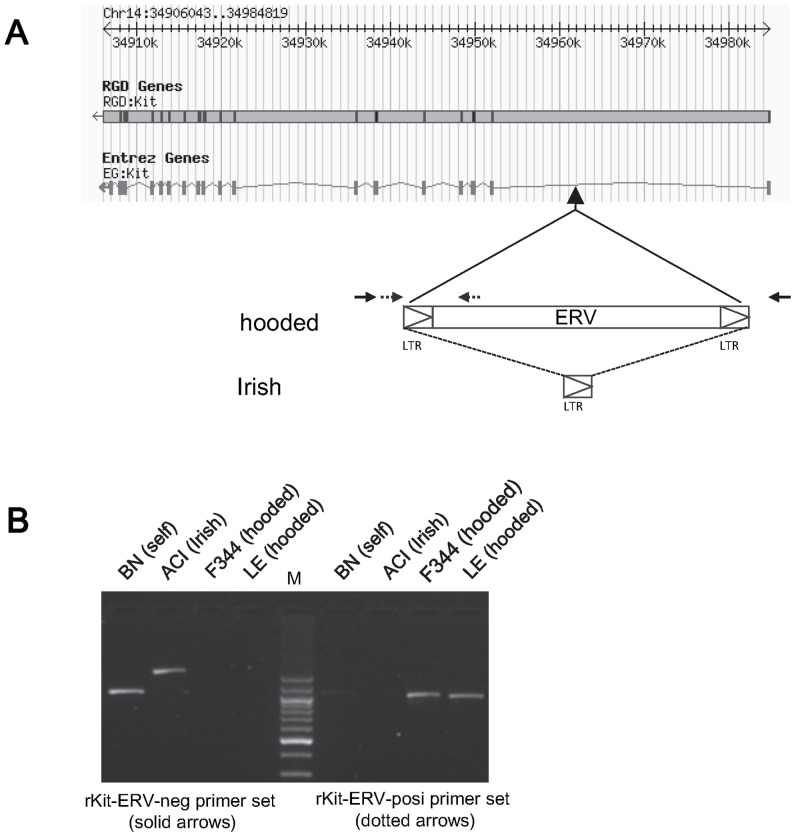
Insertion of the ERV into intron 1 of the rat Kit gene. A: Position and organization of the ERV insertion found in intron 1 of the rat Kit gene. The insertion was located 8,054 bp upstream of exon 2. The ERV sequence with 2 LTRs on both ends was found in the “hooded” strains. The solitary LTR sequence was found in the “Irish” strains. A set of primers (indicated by solid arrows) was designed to detect the absence of the ERV insertion and the presence of the solitary LTR sequence. A set of primers (indicated by dotted arrows) was designed to detect the presence of the ERV insertion. B (Left): PCR product obtained with the rKit-ERV-neg primer set (solid arrows). A 1,006 bp product was obtained from the BN (self) genome and a 1,590 pb product was obtained from the ACI (Irish) genome. Meanwhile, no products were obtained from the F344 (albino, hooded) genome and the LE (hooded) genome. (Right): PCR products obtained with the rKit-ERV-posi primer set (dotted arrows). Products 997 bp in size were obtained from the F344 genome and the LE genome, while no products were obtained from the BN genome and the ACI genome. M; 100-bp ladder molecular marker.

*De novo* sequencings of the entire inserts of the BAC clones derived from F344/Stm (*c*/*c*, *h*/*h*), and LE/Stm (*C*/*C*, *h*/*h*) rats covering the hooded critical region demonstrated that the 7,098 bp fragment was inserted into intron 1 of the rat *Kit* gene. The position of the insertion was 24,508-bp downstream of exon 1 and 8,054 bp upstream of exon 2 of the *Kit* gene. The 585-bp sequences on the both ends of the insertion showed significant similarities to the LTR of rat ERV (RLTR01_Rn) [9]. The remaining 5,930-bp sequence showed significant similarity to the rat ERV element (RAL_Rn_I) [10]. The ERV insertion was oriented on the minus strand for the *Kit* gene (**Fig. 3A**). Moreover, the BAC insert containing the Irish allele from the ACI/NJcl rat included the 584 bp LTR element only at the same position of the insertion found in the hooded allele (**Fig. 3A**).

### Genotyping of the 7,098 bp Insertion and the 584 bp Insertion

To find the correlation of the insertions with the hooded or Irish phenotype and survey the prevalence of the insertions among rat strains, we determined the presence of the insertions for 172 rat strains (**Fig. 3B**). Among 55 colored rat strains, the 18 strains with the self-coat color pattern had no insertion. The 29 strains with the hooded pattern had the ERV (7,098 bp) sequence. Finally, the eight strains with the Irish pattern had the LTR (584 bp) element only. As for the albino rat strains, all of the 117 strains had the ERV (7,098 bp) sequence, indicating the shared uniform haplotype for the albino and hooded mutations (**Table S1**).

## Discussion

The *Kit* gene encodes a cell surface receptor, c-Kit, (molecular weight 145–160 kd) that belongs to the immunoglobulin gene family and carries an intrinsic tyrosine kinase activity in its cytoplasmic portion. The interaction of c-Kit with its ligand, steel factor, leads to receptor dimerization, kinase activation, and tyrosine phosphorylation of cytoplasmic proteins. The c-Kit gene is expressed on melanocytes, gametocytes, mast cells, hematopoietic stem cells, and interstitial cells of Cajal. Thus, the Kit tyrosine kinase membrane receptor is essential for melanogenesis, gametogenesis and hematopoiesis during embryonic development and postnatal life.

c-Kit is expressed in melanoblasts during the melanogenesis starting from the time they leave the neural crest. Expression continues during embryonic development [11]. It is also expressed in the melanocytes of postnatal animals. Thus, mutations of the murine *Kit* gene manifest as dominant white spotting (*W*). In addition to the *Kit* gene, several other genes, such as *Pax3*, *Mitf*, and *Sox10*, could also be involved in the white-spotting phenotype. Mutations in these genes are related to defects of melanocyte development [12]. *W* mutations either alter the coding sequence of the Kit receptor tyrosine kinase, resulting in a receptor with impaired kinase activity, or affect *Kit* expression. *W* mutations that affect *Kit* expression are often located in the regulatory sequence. For example, the *Kit^W-57J^* allele affects the temporal and the spatial pattern of the *Kit* expression, so that *Kit^W-57J^*/*Kit^W-57J^* mice have an irregular band of spotting, lack pigmentation in their feet and tails, and have a head blaze. The *Kit^W-57J^* allele comprises an 80 kb deletion located at the 5′ end of the *Kit*-coding sequence [13]. The *Kit^W-bd^* and *Kit^W-sh^* alleles also affect the *Kit* expression pattern during the developmental stage and the both *Kit^W-bd^*/+ and *Kit^W-sh^*/+ mice show a white band in the trunk region [13], [14]. Both alleles are associated with a genomic inversions located at the 5′ region of the *Kit* gene [13]. These findings indicate that the dysregulation of the *Kit* expression affects the development of melanoblasts and thereby the white spotting appears in mutant mice.

We found the insertion of the ERV sequence in the intron 1 of the rat *Kit* gene. ERV sequences have resulted from both ancient and modern infections of exogenous retroviruses, which have successfully colonized the germ line of their host [15]. Insertion of the ERV disrupts host protein-coding genes or alters gene expression by affecting splicing or by providing novel signals for initiation, regulation or termination of transcription [15]. The ERV insertions of the first intron with antisense orientation lead the small and lower level of transcripts [16], or aberrant splicing [17], [18] in mouse mutants. Thus, we consider that the ERV insertion we found in the hooded allele may provoke the dysregulation of the *Kit* expression and thereby causes the specific hooded pattern.

We also found the solitary LTR sequence in the Irish allele. In mice, it has been known that the internal ERV sequence is deleted via homologous recombination between the 5′ and the 3′ LTRs so that a solitary LTR is left behind [19]. Such deletion reverses the mutant phenotype to the wild-type or, occasionally, attenuates the mutant phenotype. The latter case is found in the reversion of the mouse nonagouti (*a*) to the black-and-tan (*a^t^*) or the white-bellied agouti (*A^w^*). The *a* insertion consists of a 5.5-kb VL30 element that has incorporated 5.5 kb of additional sequence internally; this internal sequence is flanked by 526-bp direct repeats. Homologous recombination utilizing the 526-bp direct repeats generates the *a^t^* allele, containing only the VL30 element with a single internal 526-bp repeat. Homologous recombination utilizing the VL30 LTRs generates the *A^w^* allele, containing only a solitary LV30 LTR [16]. The rat Irish (*h^i^*) causes a white spot on the belly between and behind the front legs. Therefore, we conclude that the solitary LTR in the Irish allele also may have been generated by homologous recombination between the 5′ and 3′ LTRs of the rat ERV that is inserted in the hooded allele and that the Irish allele is a partial revertant of the hooded allele due to the residual solitary LTR.

Coat color variations have historically played important roles in genetic studies to understand the basics of inheritance. Thus, the identification of the causative mutation of the color variations followed by the survey of them for existing strains provides insights into the origin of coat color mutations. Since the coat color variations were noted in the early days of the domestication of the rat, such molecular genetic approach could give new insights on the establishment of laboratory rat strains. In the present study, we focused on the oldest color variations in the rat, albino and hooded.

We collected tissues or DNAs of rat strains from across the world in order to cover all possible albino or hooded mutations that are still available in laboratory rat strains. Thus, it appears very likely that all albino laboratory rat strains developed so far share only one common mutation: 299His in the *Tyr* gene. Most existing albino rat strains are derived from albino strains or stocks of the Wistar Institute or from crosses between Wistar albino rats and other rats including wild rats. The remaining albino rats were not directly derived from the Wistar Institute (**Table S1**). DON strains, Ihara’s rat strains, and TO strains were established in Japan, while the F344 strains and HTX strain were established in the USA, and Yagil’s rat strains were established in Israel. They also carry the same *Tyr* mutation. These findings suggest that the heredity of all albino rats can be traced back to one rat with the albino mutation.

**Figure 4:**
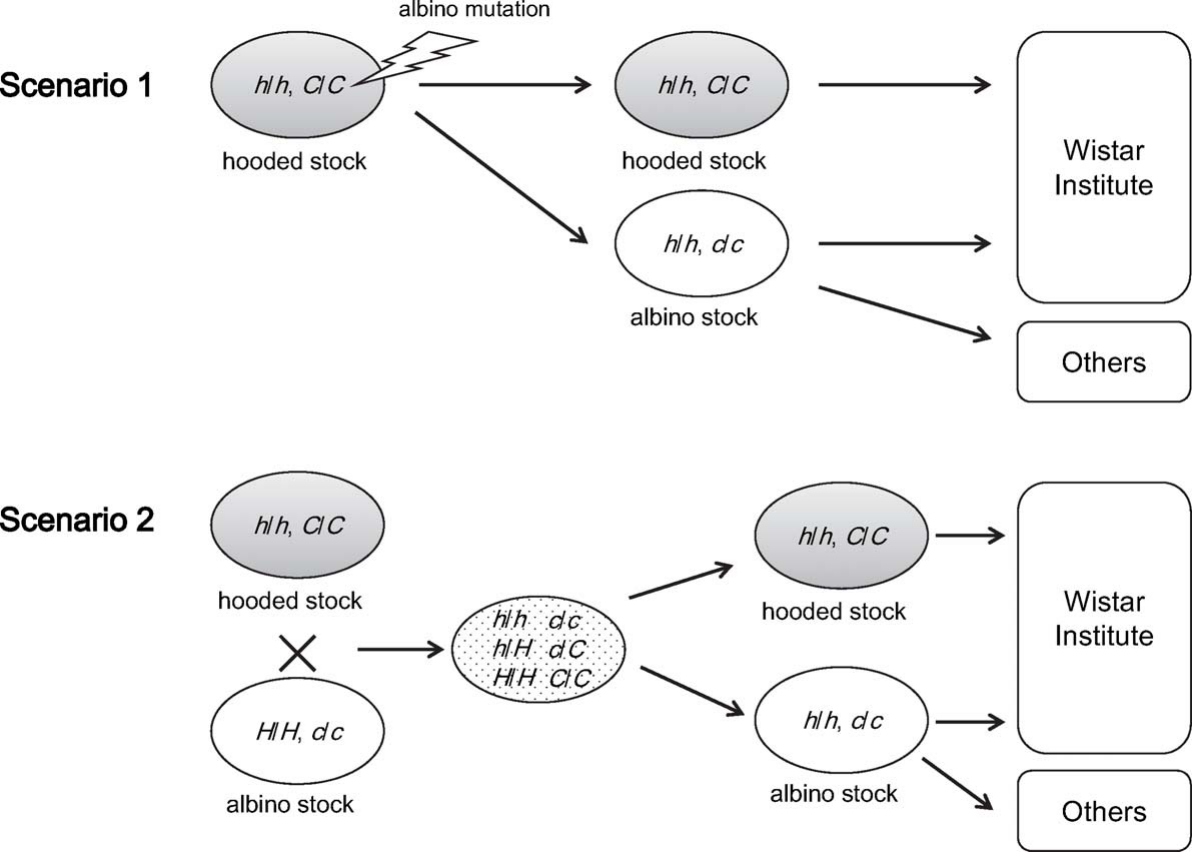
Possible scenarios for the establishment of the albino and hooded rat strains. Scenario 1: The rat albino (299His) mutation occurred in a rat of the hooded (piebald) rat stock. From the stock, the albino stock was established. Then they were introduced into the Wistar Institute and some albino rats were used for the establishment of the Wistar-independent stocks. Scenario 2: The albino stock that can be traced back to an albino rat that carried the 299His mutation and the hooded (piebald) stock were independently established. Crossing occurred between the albino and the hooded stocks. In the resultant stocks of this cross, albino rats that carried either the hooded (h) or self (H) allele must be present. The albino rats with the particular genotype (h/h, c/c) were “by chance” selected and used to establish the albino rat stock. Such new-established albino stock was introduced into the Wistar Institute and some rats from it were used for the development of the Wistar-independent albino stocks.

Moreover, we found that all albino strains we examined share the 7,098-bp ERV insertion in the *Kit* gene without any exception. According to Donaldson [4], there must have been both albino and hooded stocks before the establishment of the Wistar albino stock. Given the uniform genotypes for the albino and hooded mutations in the albino rat strains, we suggest two possible scenarios for the establishments of the albino and hooded strains. The most likely one is that the albino mutation occurred in a rat of a hooded stock. From that colony, the first albino rats were discovered and were used as founders of albino rats. Some albino rats were introduced into the Wistar Institute and some were used for developments of Wistar-independent albino stocks (**Fig. 4**). Another scenario is that albino rat strains had been developed independently of hooded stock and subsequent crossing occurred between the albino and the hooded stocks. In the resultant stocks of this cross, albino rats that carried either the hooded (*h*) or self (*H*) allele must be present. Given the evidence provided in this study, albino rats with the particular genotype (*h*/*h*, *c*/*c*) were “by chance” selected and used to establish the albino rat stock. Such newly established albino stock was introduced into the Wistar Institute and some rats were used for the development of the Wistar-independent albino stocks (**Fig. 4**). Since our survey could not detect any albino rat without the hooded ERV insertion, the second scenario appears very unlikely and is just of hypothetical nature. Therefore, it is very likely that the hooded stock had been developed earlier than the albino strains and the rat albino missense mutation occurred in one hooded rat.

## Materials and Methods

### Ethics Statement

Animal experimentation in this study was carried out in strict accordance with the Regulation on Animal Experimentations at Kyoto University. The protocol was approved by the Animal Research Committee of Kyoto University.

### Genomic DNAs

Genomic DNAs from 172 rat strains were used. They consist of 55 colored rat strains and 117 albino rat strains (**Table S1**).

### Linkage Analysis

A total of 897 backcross progeny were genotyped for the hooded (*H*) locus by identifying the appearance of the characteristic hooded mark. They consisted of 300 rats from the (ACI × WTC)F1 × WTC cross [20], 207 rats from the (BN × TM)F1 × TM cross, and 390 rats from the (BN × GRY)F1 × GRY cross [21]. All of the rats were genotyped for *D14Rat84* and *D14Got40* and rats that carried the recombinant chromosome between them were used for fine mapping of the *H* locus. Five simple sequence length polymorphism (SSLP) markers (*D14Rat13*, *D14Kur1*, *D14Kur2*, *D14Kur3*, and *D14Kur5*) were used for the fine mapping.

### Haplotype Analysis

Two hooded strains (TM/Kyo and WTC/Kyo) and four “self” strains (BDIX, BN/NSlc, DA/Slc, and IS/Kyo) were genotyped for five single nucleotide polymorphism (SNP) markers that were mapped between *D14Rat84* and *D14Got40* [22]. Their names were as follows: ENSRNOSNP2799338 (position: 34,823,877), ENSRNOSNP2799339 (34,910,749), ENSRNOSNP2799340 (34,938,580), ENSRNOSNP2799341 (35,033,604), and ENSRNOSNP2799342 (35,096,682).

### BAC Sequencing

RNB1-043O06 BAC containing the hooded allele of the rat strain F344/Stm, RNB2-428C01 BAC containing the hooded allele of the rat strain LE/Stm and NC1-272P10 BAC containing the Irish allele of the rat strain ACI/NJcl, were used for BAC sequencing. High molecular weight BAC DNAs were prepared by using a Large-Construct Kit (QIAGEN) and physically fractionated into sections several hundred base pairs in length by using a Covaris S-series (Covaris, MT, USA). The BAC inserts were sequenced by using a Genome Sequencer FLX system (Roche Diagnostics) and the sequences were assembled by using a GS *De Novo* assembler (Roche Diagnostics).

### Genotyping

The genotyping of the Arg299His missense mutation in the *Tyr* gene was performed by the PCR-RFLP analysis [5]. The PCR products amplified with a set of primers (rTyr12&13 TTTCATTCATATGTAAGTCCCTTG and GCTTAGCATTGCAAAACTCACA) were digested with *Sna*BI restriction endonuclease (New England Biolabs). In this assay, the PCR products from the wild-type (299Arg) are digested, while those from the albino-type (299His) are not digested.

To determine the presence of the ERV insertion or the LTR insertion in intron 1 of the *Kit* gene, primer sets were designed. The rKit-ERV-posi (GGCCTGTGAGTGTGAATTTG and GGACGAGCCCCCATAAATA) were used to detect the ERV insertion and the rKit-ERV-neg (ACTTAAAGACCACTGAGGACA and AATGCGGAACATCTTTCAA) were used to detect the LTR insertion.

## Supporting Information

Table S1 **Coat color phenotypes and the genotypes for Tyr and Kit genes in laboratory rat strains.**
(XLSX)
